# Association of patterns of methadone use with antiretroviral therapy discontinuation: a prospective cohort study

**DOI:** 10.1186/s12879-015-1255-7

**Published:** 2015-11-19

**Authors:** Paxton Bach, Evan Wood, Huiru Dong, Silvia Guillemi, Thomas Kerr, Julio Montaner, M-J Milloy

**Affiliations:** British Columbia Centre for Excellence in HIV/AIDS, St. Paul’s Hospital, Vancouver, BC Canada; Department of Medicine, University of British Columbia, Vancouver, BC Canada

**Keywords:** Methadone, Opiate substitution treatment, HIV, Antiretroviral therapy, Highly active

## Abstract

**Background:**

Methadone maintenance therapy (MMT) is a proven treatment strategy for opioid dependent patients. Although studies have demonstrated that MMT increases contact with the medical system and improves adherence to antiretroviral therapy (ART) in HIV-positive people who inject drugs (PWID), the effect of MMT discontinuation on ART discontinuation has not been well described.

**Methods:**

We examined the impact of continuous MMT use, MMT non-use and MMT discontinuation on the time to ART discontinuation (defined as 90 days of continuous non-use following previous enrolment) in a community-recruited prospective cohort of HIV-positive PWID followed between May 1996 and May 2013 in Vancouver, Canada. Multivariate Cox proportional hazards regression was used to examine the association between MMT use patterns and time to ART discontinuation while adjusting for socio-demographic confounders.

**Results:**

A total of 794 HIV-positive PWID were included during the study period. In an adjusted analysis, in comparison to those who were continuously on MMT, MMT non-use (Adjusted Hazard Ratio [AHR] = 1.44, 95 % Confidence Interval [CI]: 1.19–1.73) as well as discontinuing MMT (AHR = 1.82, 95 % CI: 1.27–2.60) were both found to be independently associated with time to ART discontinuation.

**Conclusions:**

This study reinforces the known benefits of MMT use on ART adherence and demonstrates how discontinuation of MMT is independently associated with an increased risk of ART cessation. These data highlight the importance of retaining PWID on MMT.

## Background

People who inject drugs (PWID) constitute a population at exceptionally high-risk for HIV infection, representing approximately 10 % of all new HIV infections in North America annually [1, 2]. In addition to an increased risk of HIV infection, PWID exhibit higher rates of disease progression and higher HIV/AIDS mortality rates as a result of increased co-morbidities and suboptimal access to antiretroviral therapy (ART) [3–6]. There is also a well-documented association between ongoing drug use and decreased ART adherence [7, 8], which can lead to the development of drug resistance [9, 10], disease progression [11, 12], and increased mortality [13]. While both ART initiation and adherence remain issues for PWID, it is encouraging to note that former PWID achieve comparable treatment outcomes as their non-PWID counterparts [14, 15], and that good results are attainable in PWID should they remain adherent to their ART regimen [16].

Methadone maintenance therapy (MMT) is an evidence-based approach to treating opioid dependence and reducing illicit opioid use [17]. Within British Columbia it is widely available through physician’s offices, health authorities, and various private services, contributing to the fact that 53.3 % of Vancouver PWID reported MMT use in 2011 [18]. In addition to its effects on opioid use, MMT is also a useful tool in combatting the spread of HIV and has been shown to reduce drug-related behaviours associated with a high risk of HIV transmission [19]. In HIV-positive PWID, MMT increases uptake and adherence to ART and improves overall treatment outcomes [20, 21]. Enrolment in MMT programs also decreases the incidence of ART discontinuation in this population [22]. Despite these benefits MMT remains inaccessible in many countries due to local drug regulations, and the five countries with the largest injection-drug driven HIV epidemics provide opioid substitution therapy to less than 2 % of their total populations of PWID [21].

While MMT has been demonstrated as an effective tool for improving ART treatment outcomes in HIV-positive PWID, many PWID will decline MMT and rates of drop out from treatment are high [23]. In this context, the impact that MMT discontinuation has on ART adherence has not been well described. The following study was conducted to test the hypothesis that discontinuation of MMT predicts a higher risk of discontinuation of ART in a long-running prospective cohort of HIV-positive PWID in Vancouver, Canada.

## Methods

This study was conducted using data from the AIDS Care Cohort to Evaluate Access to Survival Services (ACCESS), an ongoing prospective cohort study of HIV-positive PWID [24, 25]. Briefly, recruitment for the cohort occurs through street outreach in the Downtown Eastside of Vancouver, the regional epicenter for injection drug use. Participants are eligible if they are a minimum of 18 years old, are HIV seropositive, and have a history of illicit drug use. At baseline and semi-annually participants provide blood samples for virologic and serologic analysis and respond to an interviewer-administered questionnaire eliciting data on demographics, drug use patterns, and interactions with the criminal justice system. In turn, they are compensated $20 (CAD) per visit and are offered referrals to addictions treatment and other health services. Data gathered from the visits is supplemented by information on HIV treatment received from the local province-wide drug treatment program (DTP) at the British Columbia Centre for Excellence in HIV/AIDS (BC-CfE), a centralized ART dispensary and HIV laboratory for the province of British Columbia. These data include complete retrospective and prospective profiles of CD4 counts, plasma HIV-1 RNA viral loads (VL), and history of exposure to specific antiretroviral agents. The cohort receives annual ethics approval by the Providence Health Care/University of British Columbia Research Ethics Board. All individuals provide written informed consent upon study recruitment.

The study period extended from May 1996 to May 2013. Participants were eligible for inclusion if they were exposed to ART at baseline or had initiated ART during follow-up. For individuals who initiated ART during the study period, the date of ART initiation was considered their baseline date. Study eligibility required that individuals had a baseline CD4 count and viral load measured within 6 months before or after their baseline date.

The primary outcome of our study was discontinuation of ART, defined as 90 days of continuous ART non-use following previous enrolment in ART [26]. Discontinuation was determined through confidential linkage with the pharmacy database of the existing province-wide antiretroviral treatment program. The primary explanatory variable was methadone status, and participants at each study visit were categorized as either MMT non-users, continuous MMT users adherent to methadone for at least six months, or as prior MMT users who had discontinued therapy over the previous six months. This variable was time-varying, as their category could change if answers to the question “are you in a methadone treatment program right now?” were discordant between 6-month follow-up periods. Patients consistently on MMT were used as the reference category and participants were grouped based on self-reported data. Potential confounders that were considered included: gender (male vs. female), age (per 10 years older), ethnicity (Caucasian vs. other), homelessness within previous six months (yes vs. no), sex work involvement (yes vs. no), at least daily heroin injection (yes vs. no), at least daily cocaine injection (yes vs. no), at least daily crack cocaine smoking (yes vs. no), at least daily alcohol use (yes vs. no), a protease inhibitor in initial ART regimen, baseline CD4 count, baseline VL, and the number of individuals the participant’s ART-prescribing physician had experience treating at the time the participant initiated ART (previously demonstrated to affect the rate of VL suppression [27]). All behavioural variables refer to activities taking place within the previous 6 months and all variable definitions were identical to earlier ACCESS reports [24, 25].

Initially, we examined the baseline characteristics of the analytic sample stratified by methadone use. To test for significant differences we calculated Pearson’s chi-squared for categorical variables and the Wilcoxon rank-sum test for continuous variables. To estimate the bivariable relationships between each explanatory variable and the time to ART discontinuation, we used Cox proportional hazards regression. Extended multivariable Cox proportional hazards regression was then applied to examine whether methadone status was independently associated with time to ART discontinuation, after adjusting for potential confounders [28]. A confounding model selection approach was used, as previously described [29]. Briefly, we fit a multivariate model containing all variables found to be significantly associated with ART discontinuation in bivariate analyses (at *p* < 0.10). One secondary variable at a time was then removed in a stepwise manner to construct reduced models. At each step the values of the coefficients for methadone status in the full model was compared with those in the reduced models. Secondary explanatory variables corresponding to the smallest relative change were removed sequentially, and the process was continued until the minimum change from the full model was greater than 5 %. As it is an important clinical variable associated with eligibility for ART, baseline CD4 count was *a priori* retained in the final model despite non-significant results in the bivariate analysis. Finally, we describe the median HIV viral load for each methadone use category using all available data including baseline and follow-up measures.

## Results

In total, 794 HIV-positive PWID were included in the present analyses between May 1996 and May 2013, among whom 494 (62.2 %) were male and 451 (56.8 %) self-identified as Caucasian. The median follow-up time was 44.9 (interquartile range [IQR]: 24.8–78.5) months. The median age of participants at baseline was 41.1 (IQR: 34.3–46.6) years old. Table 1 displays additional socio-demographic, behavioural, social, and clinical data. When grouped by methadone status at the start time of the survival analysis, 449 participants (57 %) were not on MMT, 327 (41 %) were continuously on MMT, and 18 (2 %) had discontinued MMT within the last six months. Among 794 participants, 377 experienced a total of 766 ART discontinuation events (defined as 90 days off ART following previous enrolment), for an incidence density of 27.7 (95 % confidence interval [CI]: 25.1–30.5) per 100 person-years. Among the 794 participants, 269 (33.9 %) switched their category for MMT use during follow-up.

**Table 1:** Baseline demographics of study participants stratified by mmt use (*n =* 794)

	Methadone Status			
Variable	Total (%) (*n =* 794)	Discontinued MMT (%) (*n =* 18)	Current MMT (%) (*n =* 327)	MMT Naive (%) (*n =* 449)
Gender				
Male	494 (62.2)	9 (50.0)	166 (50.8)	319 (71.1)
Female	300 (37.8)	9 (50.0)	161 (49.2)	130 (28.9)
Age (years)				
Median (IQR)	41.1 (34.3–46.6)	37.8 (30.0–42.9)	40.6 (34.2–46.2)	41.6 (34.6–47.2)
Ethnicity				
Caucasian	451 (56.8)	12 (66.7)	205 (62.7)	234 (52.1)
Other	343 (43.2)	6 (33.3)	122 (37.3)	215 (47.9)
Homelessness^a^				
Yes	166 (20.9)	2 (11.1)	68 (20.8)	96 (21.4)
No	625 (78.8)	16 (88.9)	258 (78.9)	351 (78.2)
Sex work involvement^a^				
Yes	125 (15.7)	3 (16.7)	60 (18.4)	62 (13.8)
No	667 (84.0)	15 (83.3)	266 (81.4)	386 (86.0)
Daily heroin injection^a^				
Yes	131 (16.5)	8 (44.4)	53 (16.2)	70 (15.6)
No	662 (83.4)	10 (55.6)	274 (83.8)	378 (84.2)
Daily cocaine injection^a^				
Yes	148 (18.6)	5 (27.8)	57 (17.4)	86 (19.2)
No	640 (80.6)	13 (72.2)	268 (82.0)	359 (80.0)
Daily crack cocaine smoking^a^				
Yes	208 (26.2)	4 (22.2)	104 (31.8)	100 (22.3)
No	585 (73.7)	14 (77.8)	222 (67.9)	349 (77.7)
Protease inhibitor initial regimen^a^				
Yes	325 (40.9)	11 (61.1)	143 (43.7)	171 (38.1)
No	469 (59.1)	7 (38.9)	184 (56.3)	278 (61.9)
CD4 count (cells/mm^3^)				
Median (IQR)	300 (190–440)	255 (190–310)	320 (180–470)	295 (190–425)
Viral load (log_10_ copies/mL)				
Median (IQR)	3.7 (1.7–4.7)	4.5 (4.0–4.8)	3.5 (1.7–4.7)	3.8 (1.7–4.8)
Physician HIV experience^b^				
Median (IQR)	60 (14–161)	133 (34–203)	71 (19–185)	52 (11–139)

^a^Refers to behaviors in the previous six months. ^b^ Refers to number of total HIV patients physician has treated prior to initial visit with participant. IQR = inter-quartile range. Percentages may not sum up to 100 % due to missing data and/or rounding error

Table 2 shows results of the bivariate and multivariate Cox regression analyses of time to ART discontinuation. In the bivariate analyses, both being continuously off MMT (hazard ratio [HR] = 1.36, 95 % CI: 1.12–1.65) as well as discontinuing methadone (HR = 3.03, 95 % CI: 2.12–4.33) were associated with an increased risk of discontinuing ART when compared to consistent MMT use. Additionally risk factors included homelessness within the previous six months (HR = 1.94, 95 % CI: 1.57–2.39), sex work involvement (HR = 1.64, 95 % CI: 1.28–2.10), at least daily heroin injection (HR = 2.59, 95 % CI, 2.09–3.21), at least daily cocaine injection (HR = 2.10, 95 % CI: 1.72–2.58), at least daily crack cocaine smoking (HR = 1.40, 95 % CI: 1.17–1.68), at least daily alcohol use (HR = 1.63, 95 % CI: 1.27–2.09), and a higher baseline VL (per log_10_ copies/mL; HR = 1.49, 95 % CI: 1.40–1.58). Male gender (HR = 0.71, 95 % CI: 0.58–0.86), older age (per 10 year increase; HR = 0.49, 95 % CI: 0.43–0.55), Caucasian ethnicity (HR = 0.78, 95 % CI: 0.64–0.95), and baseline CD4 count (per 100 cells/mm^3^; HR = 0.95, 95 % CI: 0.90–0.99) were negatively associated with ART discontinuation.

**Table 2:** Bivariate and multivariate cox proportional hazards analyses of factors associated with art discontinuation among a sample of injection drug users in vancouver, canada (*n =* 794)

	Unadjusted Hazard Ratio (HR)			Adjusted Hazard Ratio (AHR)		
Variable	HR	95 % CI	*p* value	AHR	95 % CI	*p* value
MMT						
Current MMT	1.00	---	---	1.00	---	---
Not on MMT	1.36	1.12–1.65	0.002	1.44	1.19–1.73	<0.001
Discontinued MMT	3.03	2.12–4.33	<0.001	1.82	1.27–2.60	0.001
Gender						
(Male vs. female)	0.71	0.58–0.86	<0.001	---	---	---
Age						
(Per 10 years older)	0.49	0.43–0.55	<0.001	0.59	0.52–0.66	<0.001
Ethnicity						
(Caucasian vs. other)	0.78	0.64–0.95	0.015	---	---	---
Homelessness^a^						
(Yes vs. no)	1.94	1.57–2.39	<0.001	1.53	1.24–1.87	<0.001
Sex work involvement^a^						
(Yes vs. no)	1.64	1.28–2.10	<0.001	---	---	---
Daily heroin injection^a^						
(Yes vs. no)	2.59	2.09–3.21	<0.001	1.70	1.39–2.08	<0.001
Daily cocaine injection^a^						
(Yes vs. no)	2.10	1.72–2.58	<0.001	---	---	---
Daily crack cocaine smoking^a^						
(Yes vs. no)	1.40	1.17–1.68	<0.001	---	---	---
Daily alcohol use						
(Yes vs. no)	1.63	1.27–2.09	<0.001	---	---	---
Protease inhibitor initial regimen^a^						
(Yes vs. no)	0.97	0.79–1.19	0.771	---	---	---
Baseline CD4 count						
(Per 100 cells/mm^3^)	0.95	0.90–0.99	0.025	1.03	0.98–1.07	0.234
Baseline viral load						
(Per log_10_ copies/mL)	1.49	1.40–1.58	<0.001	1.39	1.29–1.50	<0.001
Physician HIV experience^b^						
(Per # prev. patients)	1.00	1.00–1.00	0.074	---	---	---

^a^Refers to behaviors in the previous six months. ^b^Refers to number of total HIV patients physician has treated prior to initial visit with participant

In the multivariate analysis, both being continuously off MMT (adjusted hazard ratio (AHR) = 1.44, 95 % CI: 1.19–1.73) and discontinuing MMT (AHR = 1.82, 95 % CI: 1.27–2.60) were each independently associated with an increased risk of discontinuing ART.

When we looked at the median plasma HIV viral load throughout study period, the median viral load was less than 50 (IQR: 35–1005) copies/mL among individuals where MMT was used continuously and was 1482 (IQR: 49–39099) copies/mL among individuals who were on but discontinued MMT in the prior six months.

As a subanalyis, we investigated all possible interactions between engagement in methadone and all other explanatory variables significant in the final multivariable model. Of these, neither MMT * age (*p* = 0.085) nor MMT * homelessness (*p* = 0.315) were significantly associated with the outcome. The interaction terms for MMT * ≥ daily heroin injection (*p* = 0.008) and MMT * baseline VL (*p* = 0.012) were significant. In light of these findings, we fit stratified models to estimate MMT effects by each level of heroin injection. Among individuals < daily heroin injection, not being on MMT was associated with ART discontinuation (AHR = 1.30, 95 % CI: 1.05–1.60) while discontinuing MMT was not associated with ART discontinuation (AHR = 1.45, 95 % CI: 0.90–2.34). Among individuals with ≥ daily heroin injection, both not being on methadone (AHR = 2.38, 95 % CI: 1.60–3.52) and MMT discontinuation was associated with ART discontinuation (AHR = 3.05, 95 % CI: 1.80–5.19).

## Discussion

In this study we examined the relationship with being off of MMT as well as with discontinuing MMT on ART discontinuation. Our analysis revealed that participants not engaged in MMT have a higher likelihood of ART discontinuation, and additionally demonstrated the independent association of MMT discontinuation reducing time to ART discontinuation. Despite MMT discontinuation occurring within only the previous six-month period, it is notable that the median VL of these participants was higher than those who remained on MMT.

Multiple behavioural, social, and structural barriers complicate the treatment of HIV infection among PWID [21, 30]. In this setting, it is well documented that ongoing injection drug use contributes to poor ART adherence, and as a result poor health outcomes [7, 8, 13]. MMT has been shown to mitigate these outcomes in opioid-dependant PWID by enabling initiation of HIV treatment and by improving ART adherence [20–22]. Our data add further support to the positive effects of MMT on optimizing HIV/AIDS treatment outcomes, while providing evidence suggesting that MMT discontinuation is independently associated with ART discontinuation. This is consistent with studies demonstrating that other benefits of MMT are improved with extended duration of methadone treatment including decreased relapse into drug use, reduction in illegal activity, and increased full-time employment [31, 32]. Interestingly, the negative consequences of forced discontinuation of MMT have recently been hypothesized following the Russian occupation of Crimea and a resulting reversal in government policy previously supportive of MMT [33]. Our data lends support to the suggestion that such policy changes could have significant negative impacts on HIV/AIDS treatment programs in affected regions.

The mechanisms through which remaining on MMT decreases the discontinuation of ART are likely multifactorial. MMT provides a stabilizing effect on opioid use, encouraging improved social function, social support, and development of daily routines [34]. These have a demonstrated positive effect on ART adherence [35]. Furthermore, MMT necessitates regular contact with the medical system, allowing for the establishment of positive patient-provider relationships, providing opportunities for the management of psychiatric co-morbidities and medication side effects, and offering regular access to counselling and other programs. These are all factors understood to improve ART adherence in PWID [35].

In response to the beneficial effects of MMT on ART enrolment and adherence, co-administration has been studied in multiple models of directly observed therapy (DOT) or directly administered antiretroviral therapy (DAART) [36, 37]. In our setting, ART is regularly co-administered with MMT, which can be prescribed through community physician’s offices and dispensed by community pharmacies for witnessed ingestion. A recent systematic review identified these programs as successful adherence interventions for HIV-positive PWID in the short-term [36], while a second meta-analysis also observed a positive effect on virologic, immunologic, and adherence outcomes, particularly when they target individuals with a high risk of non-adherence [37]. Of note, both studies comment on the need for further evaluation of the long-term benefits of these interventions as initial intervention effects appeared to wane following completion of the programs. These results are congruent with our work, demonstrating that short-term interventions are less effective at improving ART adherence over time. Our data is further complemented by the recent observation that act of enrolling in MMT decreases the risk of ART discontinuation [22], suggesting clearly that long-term treatment is necessary to maximize the benefits of MMT on ART adherence.

This study has several limitations, in part due to its nature as an observational cohort study. While our cohort is largely representative of the local population of PWID, the generalizability of our findings to other settings is not known. Additionally, much of our data relies on self-reporting of sensitive subjects and there is a possibility of a response bias in our survey results. Third, data on reasons for discontinuation was not available. This information could potentially provide further information as to which patients are at higher risk, including those taking particularly complicated ART regimens or those experiencing significant side effects, and might be best explored through a future qualitative study. Fourth, as our study does not include diagnostic screening for opioid dependence, some misclassification bias may have been introduced by including individuals not eligible for MMT. We did, however, include various measures of illicit drug use in this analysis, and our model-building protocol indicated that high-intensity cocaine and crack use did not substantively affect the relationship between MMT engagement and time to ART discontinuation. Finally, as this is an observational study we must acknowledge the possibility that the described relationship between MMT and ART discontinuation is not a causal one. Factors with known effects on ART adherence not included in our survey include the presence of psychiatric co-morbidities and treatments and the strength of social supports, which we were unable to incorporate into our analysis [38]. Despite these limitations, the data suggesting that MMT plays a positive role in successfully maintaining patients on ART continues to grow.

## Conclusions

This study provides evidence that discontinuing MMT is independently associated with an increased risk of discontinuing ART. These data offer further support for MMT as an important component of comprehensive HIV treatment programs, and highlight the need for evidence-based strategies for retaining PWID on MMT.

## Abbreviations

ART: Antiretroviral therapy
DAART: Directly administered antiretroviral therapy
DOT: Directly observed therapy
DTP: Drug-treatment programs
MMT: Methadone maintenance therapy
PWID: People who inject drugs
VL: Viral load
